# Virtual Reality for Pain Management During Repeated Pediatric Laser Procedures: Protocol for a Pilot Randomized Clinical Trial

**DOI:** 10.2196/87207

**Published:** 2026-06-09

**Authors:** Megan Armstrong, Hannah Williams, Esteban Fernandez Faith, Ai Ni, Henry Xiang

**Affiliations:** 1 Center for Injury Research and Policy, Abigail Wexner Research Institute, Nationwide Children's Hospital, 700 Children's Drive, RB3, Columbus, OH, 43205, United States, 1 614-355-5890; 2 Center for Pediatric Trauma Research, Abigail Wexner Research Institute, Nationwide Children's Hospital, Columbus, OH, United States; 3 College of Medicine, The Ohio State University, Columbus, OH, United States; 4 Division of Dermatology, Nationwide Children's Hospital, Columbus, OH, United States; 5 Division of Biostatistics, College of Public Health, The Ohio State University, Columbus, OH, United States

**Keywords:** laser, virtual reality, VR, dermatology, pediatric, anxiety, pain, distraction, CO_2_, pulsed dye laser, PDL

## Abstract

**Background:**

Lasers have wide applications in medicine but are associated with pain and anxiety, particularly in younger patients. Pain mitigation is often limited to topical anesthetics in the outpatient setting. Distraction techniques are limited by the need for ocular protection, which can include eye patches that completely occlude vision. Virtual reality (VR) is effective at managing procedural pain and anxiety during other short medical procedures and is a promising tool for this population.

**Objective:**

This trial aims to assess the safety, feasibility, and efficacy of the virtual reality pain alleviation therapeutic (VR-PAT) for pain management during outpatient laser procedures.

**Methods:**

A total of 40 patients requiring outpatient laser therapy for at least 2 sessions will be recruited from a pediatric hospital in the Midwestern United States for this crossover randomized, 2-arm clinical trial. During the first laser visit, the participants will be randomly assigned to either play the VR-PAT game during their procedure or wear the headset with a dark screen. Participants will answer questions about their pain (Numeric Rating Scale 0‐10), anxiety (State-Trait Anxiety Inventory for Children, Numeric Rating Scale 0‐10, and Modified Yale Preoperative Anxiety Scale), and pain medication usage. Those playing the VR-PAT will report simulator sickness symptoms and their experience playing the game. At their second laser visit, participants will cross over to the opposite intervention. The primary outcomes are the differences in self-reported pain and anxiety between the 2 interventions. Feasibility outcomes include the proportion of screened patients who were eligible, have given consent, and completed both visits, as well as adverse events reported. To evaluate the efficacy of pain reduction, composite pain scores, and pain medication usage will be calculated for each laser visit. To evaluate the efficacy of anxiety reduction, the change in Modified Yale Preoperative Anxiety Scale scores will be compared between control and VR groups at each visit using the Wilcoxon rank sum tests. All statistical analyses will follow the intention-to-treat principle with regard to intervention assignment at each visit.

**Results:**

The study was funded in January 2023 and began enrollment at that time. A total of 44 participants were recruited, and data collection was completed in November 2025, with 40 participants completing both visits. The sample was balanced, with 40 participants using the intervention and participating in the control condition. The age range of the complete sample was 6 to 21 years at recruitment, and 22 (55%) were female. Data analysis is in progress with final results planned for June 2026.

**Conclusions:**

Findings from this innovative randomized clinical trial will provide early evidence on the efficacy of the VR-PAT in reducing self-reported pain and anxiety during outpatient laser procedures. The results from this trial will inform a large-scale, multisite study.

## Introduction

### Background and Significance

Lasers have wide applications in medicine and dermatology. In pediatric patients, laser technology is often used to treat congenital and acquired skin conditions. Pulsed dye laser (PDL) is the standard of care for the treatment of vascular birthmarks, primarily port-wine stains and capillary malformations. This laser is also used to treat infantile hemangiomas (including residual changes and ulceration), spider angiomas, and pyogenic granulomas. Additionally, lasers have become a critical component in the treatment of scars resulting from burns, trauma, surgery, and inflammatory conditions. Apart from vascular lasers, such as PDL, ablative lasers, such as the fractional CO_2_ laser, are used to improve the size, texture, symptoms, and functional limitations caused by scars.

Though it is an effective and important treatment modality in pediatric dermatology, laser therapy is often associated with significant pain for the patient [1,2]. Pain associated with laser procedures is typically described as a heat or burning sensation. PDL is described as feeling like a hot rubber band snapping on the skin with each pulse [3], and the laser pulses 15 to 100 times per treatment session [4]. Infants and young children may require some form of anesthesia [3], and even adolescents and adults may have difficulty tolerating the procedure [5].

It is vital to mitigate pain and anxiety during laser procedures in pediatric patients. Unrelieved pain during pediatric procedures has been associated with adverse effects on recovery and postoperative behavioral abnormalities [6-8], including postprocedural symptoms of posttraumatic stress disorder [9]. Anxiety and pain can lead to a lack of cooperation with the procedure by the patient, which may impact the effectiveness of the therapy [5]. Darkness from laser eye protection and pain-related distress may also precipitate phobic responses in the patient [4], which increases their anxiety and can negatively impact subsequent treatments [10], as well as future interactions in health care settings [11]. Experiences of undertreated pain can have significant adverse effects on immediate and long-term mental health for pediatric patients [12]. Therefore, effective pain mitigation during pediatric laser procedures is a crucial component of biobehavioral intervention targeting pediatric patients.

Pharmacologic analgesia is often the primary pain management technique during pediatric laser procedures. Topical anesthetics, often lidocaine, are the most commonly chosen analgesics during pediatric laser procedures [13]. Topical agents are generally well-tolerated in the pediatric population and can reduce pain associated with laser procedures, but they are often insufficient for pain mitigation in all patients [3]. Additionally, topical agents may not be a viable option when the lesion involves large regions of the body or is located near the ears, fingers, eyes, or nose [2]. Local injectable lidocaine is an additional pain relief option. However, epinephrine is typically included in the injected solution, which may decrease lesion size and hinder the effectiveness of the laser treatment for vascular lesions [3]. Furthermore, the use of a needle may result in increased pain or discomfort, lesion distortion, and there may be concern for lidocaine toxicity if the anesthetized area is large [5]. General anesthesia is an option that is occasionally used [13], and it can be especially useful for children who are highly uncooperative or distressed [3]. However, this option is very carefully considered due to growing concern for neurocognitive defects resulting from the use of general anesthesia in children [13,14]. Sedatives, often midazolam, can be administered to alleviate both pain and anxiety, as well as to induce anterograde amnesia [5]. Unfortunately, midazolam is associated with a primary side effect of respiratory depression, which is a serious concern [3].

It is preferable to treat dermatologic conditions as early as possible [5]; the response rate to laser procedures is significantly better in younger children [15], and delaying the procedure may negatively impact psychosocial development due to exposure to social stigma [4]. Therefore, waiting until the child has aged to maximize tolerance of the laser procedure is not ideal. Efforts must be made to minimize the pain and anxiety associated with the laser procedure for the pediatric patient.

Ocular exposure to laser radiation can result in permanent eye damage, including vision loss [5]. It is paramount that any eye protection used during the procedure effectively blocks radiation exposure to the patient’s eyes. A variety of eye protection options can be used. Eye patches fully cover the eyes and thus immerse the patient in darkness, which can induce anxiety during these laser procedures. The special eye protection needed by patients during laser treatment creates a unique opportunity to use a virtual reality (VR) headset not only as eye protection but also as a pain and anxiety management strategy.

### Gap in Knowledge

There is a clear need for the development of alternative nonpharmacologic pain and anxiety relief modalities for pediatric procedural pain. Recently, distraction has emerged as a highly effective pain-mitigation tool for this population. According to the cognitive-affective model of pain, distraction alleviates pain by consuming attentional resources that would otherwise be devoted to pain processing [16], thereby decreasing subjective pain and related distress [17]. Various forms of distraction have been found to be effective at reducing subjective pain [10,18], decreasing pain-related brain activity [19], and increasing positive procedural outcomes [20] in pediatric populations.

Among the various forms of distraction, VR has been found to be highly effective in medical settings for pediatric patients. VR uses a head-mounted display to create a highly immersive environment that blocks the sights and sounds of the hospital and can distract the user from real-world pain [11]. VR has been found to be significantly better than the standard of care at decreasing self-reported pain [21-23], anxiety [10,24], time spent thinking about pain [10,23], heart rate [18], and pain-related brain activity [25,26] during painful pediatric procedures. VR has also been found to improve cognitive and emotional processing of the event [18], and VR use reduces the formation of phobic responses to hospital environments [27].

The vast majority of these studies reported no instances of simulator sickness or other immediate adverse effects of using VR [10,25,28] and noted that the majority of patients found the VR experience to be enjoyable [23,28]. In addition, both parents and nurses tend to be satisfied with the experience of using VR on the patient [29], and nurses have reported that children are more cooperative during procedures while using VR, which improves the efficacy of the procedure itself [12]. Additionally, the effectiveness of VR-based pain relief does not seem to diminish when used repeatedly, and patients continue to find the technology fun to use [23,27]. VR is a promising anxiety- and pain-relief modality that is becoming increasingly flexible, immersive, and affordable, making it easy to use in medical settings and highly applicable to a wide variety of patients and medical settings [28].

Despite extensive research regarding VR use in medical settings, there remains a knowledge gap regarding the efficacy and safety of VR in pediatric laser procedures. Most previous studies were conducted in the context of burn wound dressing changes, IV placement, or laboratory-induced pain. We are aware of very few studies examining the use of VR in the setting of laser procedures. Jaquez et al [30] found promising early results for nonpharmacologic distraction during a variety of dermatologic procedures, but this was not exclusive to PDL or CO_2_ lasers. More research is needed to understand the potential application of VR in this setting. Additionally, little is known about the safety of using VR headsets during laser procedures. Ocular exposure to laser radiation can result in permanent eye damage, including vision loss [5]. It is paramount that any eye protection used during the procedure effectively blocks radiation exposure to the patient’s eyes.

### Prior Work

Our previous clinical study provided strong evidence regarding the efficacy of the smartphone version of the virtual reality pain alleviation therapeutics (VR-PAT) for significant pain reduction during burn dressing changes [29]. This randomized clinical trial (RCT) tested the efficacy and feasibility of this tool among 90 pediatric outpatient patients with burn injuries (aged 6 to 17 years). Participants were randomly assigned to an active VR-PAT group (n=31), a passive VR-PAT group (n=30), or a standard care group (n=29) during a burn dressing change. Active VR-PAT significantly reduced observed and self-reported pain during burn dressing changes. Patients and caregivers reported satisfaction with the VR-PAT, and nurses reported that the tool could be easily used in clinics.

In another RCT study, we examined the efficacy of VR-PAT as a pain alleviation tool during at-home dressing changes for pediatric patients with burn injuries (aged 5‐17 y) [31]. Participants (n=35) were randomly assigned to either the active VR-PAT condition or the control condition. Participants found VR-PAT to be a useful distraction during home dressing changes and reported it to be easy to implement. In the VR-PAT group, children and caregivers reported that pain decreased as the week of dressing changes progressed and was lower than that in the control group after the fourth dressing change. Children playing VR-PAT reported consistent happiness and fun as the week went on, alongside increased realism and engagement, indicating that repeated use of the tool does not diminish its efficacy.

Our previous studies and preliminary results position us well to expand the VR-PAT for anxiety relief and pain reduction during pediatric laser therapy.

### Objectives

The overall objective of this proposal is to conduct a randomized crossover clinical trial to assess the safety, feasibility, and efficacy of VR-PAT for pain management during outpatient laser procedures and to evaluate anxiety as a preprocedural key factor for pain during these procedures. Our specific aims are to (1) assess the feasibility and efficacy of VR use in reducing pain in patients undergoing dermatologic laser procedures, and (2) evaluate the impact of VR use on reducing anxiety in patients undergoing dermatologic laser procedures. Our central hypothesis is that VR-PAT is safe to use and can effectively and significantly reduce pain during pediatric dermatologic laser procedures.

## Methods

### Trial Design

We will conduct a prospective, 2-group crossover RCT with a 1:1 allocation ratio to either the intervention (VR-PAT gameplay) or the control (VR headset turned off) for the first laser appointment. Participants will crossover to the opposite group at their second laser appointment. Pain, anxiety, and VR engagement (for those in the intervention group) will be assessed after each research appointment, spaced approximately 6 to 8 weeks apart. The findings will be reported based on the CONSORT (Consolidated Standards of Reporting Trials) guidelines. This protocol is reported in accordance with the SPIRIT (Standard Protocol Items: Recommendations for Interventional Trials) guidelines (Checklist 1).

### Settings and Participants

Participants will be enrolled from the outpatient dermatology clinic on the main campus of Nationwide Children’s Hospital (NCH) in Columbus, Ohio. We plan to recruit 40 patients (aged ≥5 y) to complete 2 laser visits who clinically require laser therapy. The inclusion criteria are: (1) dermatology patients (≥5 y) undergoing the first of a new series of laser procedures at the NCH Outpatient Dermatology Clinic, (2) having a legal guardian present for patients aged less than 18 years during the procedure (for informed consent), and (3) the ability to communicate orally. Exclusion criteria include: (1) any wounds that may interfere with study procedures; (2) use of the diode laser; (3) vision, hearing, or cognitive and motor impairments preventing valid administration of study measures; (4) history of motion sickness, seizure disorder, dizziness, or migraine headaches precipitated by visual auras; (5) minors in foster care; (6) inability to communicate in English; (7) pregnant women; or (8) prisoners. The exclusion of diode laser use was determined during safety testing, in which laser safety was confirmed for PDL and CO_2_ lasers but not diode lasers. Laser safety testing was performed by a third-party company to evaluate the VR headset for ocular safety. They determined that the headset was safe to use with PDL and CO_2_ lasers but did not provide protection against diode laser wavelengths. Based on the previous studies, patients aged less than 5 years were unable to respond effectively to pain measurement and other study questionnaires.

The laser team (led by our physician champion) will identify potential study individuals, and our research associates will screen them for eligibility. Once a patient’s eligibility is confirmed, a trained researcher will approach the patient and their legal guardian (if necessary) to introduce the study. Trained research staff will ensure participants are awake, alert, and able to provide assent (if the child is >9 y but <18 y) or consent if aged ≥18 years, and demonstrate understanding of the study before being consented. According to the NCH institutional review board (IRB) policy, patients aged less than 9 years only need consent from a caregiver who is the legal guardian when the study involves only minimal risk; however, all participants will be asked for verbal assent. Signed consent and assent (if applicable) will be obtained before formally enrolling patients into the study. During the informed consent conversation, the legal guardian and the patient (if aged ≥18 y) will be asked for permission to store the participant’s protected health information and identifiable information for future IRB-approved research. The person providing consent can opt out of this option, which will not affect their standard of care treatment. Biological specimens will not be collected for this study.

### Participant Timeline

Study enrollment at NCH—patients will be recruited prior to their first sequential dermatologic laser procedure. After consent and assent (if applicable) are obtained, patients will be randomized to either the VR-PAT intervention or the control group. A research associate will collect background information, such as demographics, anxiety levels, and prior video game experience, before randomization. VR-PAT instructions will be provided to the intervention group prior to participation.

Laser procedures at NCH (n=2 visits)—patients will receive their laser procedure as usual. Participants in the intervention group will wear the Pico headset and play the VR-PAT during their procedure. Participants in the control group will receive their laser procedure while wearing the VR headset (without the game). A researcher in the laser treatment room will observe the procedure and document, for the intervention group, the amount of time spent playing the VR-PAT, whether the participant declined to use it, and the number of interruptions of VR. Following the procedure, a different researcher (blinded to the group) will ask both groups of participants about their pain experience and satisfaction with the game. This process will be repeated once for each participant, as they will cross-over to the opposite intervention group at their next laser procedure.

### Randomization and Blinding

A block randomization scheme with a block size of 4 will allocate participants to 1 of the 2 intervention sequences (VR-PAT followed by control or control followed by VR-PAT) at a 1:1 ratio without stratification. We chose a block randomization strategy to avoid imbalances in allocation to the intervention at the first visit in case we were unable to recruit our planned sample. The randomization scheme was developed and uploaded to a study-specific REDCap (Research Electronic Data Capture; Vanderbilt University) project by the project manager. Study participants are randomized into intervention sequences after signing consent and completing the baseline survey. Research recruiters and participants are not aware of the intervention assignment until the participant is randomized in REDCap.

We cannot blind the participants or the clinical team to the assigned intervention since the headset will either be turned on or off. The researcher who randomized the participant and observed the laser procedure will not be blinded, but a second researcher, who is blinded to the intervention group, will collect pain ratings and anxiety post procedure. The second researcher will wait in a different room during randomization and the laser procedure, and the first researcher will remind the participant at the end of the laser procedure to keep their group a secret until they are asked. The study biostatistician will also be blinded to the intervention group while performing analyses on the primary outcome. The data will be blinded by removing labels such as “VR” and “Control” and replacing them with unidentifiable letters (eg, “A” and “B”). The biostatistician will need to be unblinded for secondary outcomes, as only participants in the intervention group will answer the VR experience questions.

The blinded researcher will need to be unblinded after pain and anxiety ratings are obtained, as those in the control environment will not be able to answer the VR experience questions. The researcher will become unblinded by asking participants whether they played the game. The biostatistician will also be unblinded for full analyses by being provided with the full dataset, including complete data labels and VR questions. This unblinding will take place after the primary outcome analysis has been completed.

### Intervention

VR-PAT is the intervention for this RCT. VR-PAT is a standalone mobile health tool developed at NCH and does not require an internet or WiFi connection. The intervention is hosted on a Pico Neo 3 Pro Eye, which is a standalone VR headset that supports native eye tracking, enabling applications to gather real-time user feedback for behavior-based application control. Our VR game is the only application available on the headset and starts immediately upon pressing the power button, which ensures all participants play the VR game intended for this study.

During the laser procedure, participants will play the virtual game by slightly tilting their heads, thereby minimizing interference with the procedure while cruising on a boat and aiming for the snow-blowing statues floating along the riverbank. The statues will burst and emit snow if the participant correctly aims at them, and a thermometer placed at the front of the boat will show decreased temperatures as more snowflakes are blown. As feedback to reinforce continued engagement, a scoreboard placed beside the thermometer will show participants the number of statues they have activated. Additionally, as the temperature drops, snow and ice will start piling up on the boat and its surroundings, providing an enhanced “cooling” experience for dermatology patients. Audio from the headset provides surrounding and directionally-adaptive effects to match the progress of the game and the direction in which a participant’s head is turning, further enhancing the immersive experience of VR-PAT during dermatologic laser procedures. The active VR-PAT game can last indefinitely, so it can be used for the entire procedure without interruption.

Participants playing the VR will be advised to discontinue using the headset during a procedure if it causes motion sickness, headaches, nausea, or dizziness. The dermatologist will pause the laser procedure and switch the eye protection to one available in the outpatient clinic. Any adverse events resulting from participation will be noted and reported in the participant surveys.

The comparator, or control, for this study is the same VR headset without the game, displaying only a dark screen. This comparator was chosen to maintain the experience of wearing something on the face for both groups. Patients undergoing a laser procedure are required to wear eye protection, which can include adhesive eye patches that occlude the eyes and create a similarly dark environment.

Participants in either intervention will be allowed to use any pain or anxiety medication that the dermatologist deems appropriate. Any medications provided will be documented.

### Measures

All data will be entered directly into the REDCap database as it is collected. A second researcher will check the REDCap records after each visit, ensuring the completeness and accuracy of the data. Data collected for this study include participant data, legal guardian data, nurse data, observed measures, and medical record review.

#### Participant Data (Self-Reported)

Prior to randomization, participants will report their prior video game experience, including the type of video games played, frequency, and favorite games. Before the laser procedure, all participants (control and intervention visits) will self-report their anxiety state using the State-Trait Anxiety Inventory for Children [32] (ratings ranging from “not at all” to “very much”) and their current anxiety using the Numeric Rating Scale (NRS; 0‐10) [33]. After the procedure, the participants will answer the remaining survey questions assessing overall pain score (NRS 0‐10) [34,35], worst pain score (NRS 0‐10), time spent thinking about pain (NRS 0‐10), length of the procedure (in minutes), and anxiety during the laser procedure (NRS 0‐10). Those playing the VR-PAT game will report their experience with the VR game (degree of realism, fun, engagement, and satisfaction; NRS 0-10) and respond to qualitative questions about their preferences with the VR-PAT. Participants will also be asked to report any simulator sickness associated with the VR game and whether they would want to use the VR again for future laser visits outside of research. These data will be collected at each of the 2 laser procedure study visits.

#### Legal Guardian Data (Self-Reported when Participants are <18 y)

Before the laser procedure, all legal guardians will self-report their anxiety state using the short-form 6-item State-Trait Anxiety Inventory [36], with ratings ranging from “not at all” to “very much”. After the procedure, the legal guardian will report their perception of the participant’s pain by providing an overall pain score (NRS 0‐10) and a worst pain score (NRS 0‐10). These data will be collected at each of the 2 study visits.

#### Nurse Data (Self-Reported)

To assess the practicality and utility of the VR experience as a pain and anxiety management tool for dermatologic laser procedures, the nurses will report whether the distraction tool is helpful and whether it is easy to use during laser procedures (yes or no).

#### Observed Measures (Research Collected)

The Modified Yale Preoperative Anxiety Scale (mYPAS) [37] will be assessed at each laser procedure before entering the treatment room, before the procedure begins, and immediately following the procedure. This is a 5-item scale, with a score of more than 30 indicating anxiety. During the procedure, the researcher will document the start and stop times of the laser procedure, the start and stop times of the VR (if applicable), whether the participant declined to use the VR-PAT, and the number of voluntary interruptions of the VR-PAT.

#### Medical Record Review (Research Abstracted)

The researcher will review the participant’s medical record and document any pain-related medication (name and dosage) that the participant used prior to the laser procedure. Demographics and procedure information recorded will include the date of birth, sex, race, ethnicity, and dermatologic variables (laser type, diagnosis, lesion size, lesion type, and lesion location).

### Outcomes

#### Feasibility Outcomes

The feasibility of using VR during outpatient laser procedures will be evaluated by the proportion (%) of patients screened who are eligible to be approached, those who consent to the study, and those who complete both study visits. This will be tracked in our screening log and analyzed periodically throughout the study for funding reporting. Adverse events, including simulator sickness, will also be used to evaluate feasibility (see “Adverse Events and Oversight” section for specifics). The threshold for scaling up this study is no serious adverse events reported, less than 10% loss to follow-up due to the VR, and greater than 80% of both patients and nurses being satisfied with the intervention. Clinical loss to follow-up will not be counted in this measure. There is no predefined proportion for recruitment and consent, but these outcomes will be used to determine the possible sample size.

#### Primary Outcomes

Difference in self-reported worst pain, average pain, and time spent thinking about pain (NRS 0 [min]−10 [max]) between VR-PAT and control visits. These questions are asked at each visit and will be compared between the intervention and control visits.

Change in procedural anxiety (mYPAS observed by the researcher, 23.33 [min]−100 [max], with higher scores denoting higher levels of anxiety) is assessed at 3 time points: prior to entering the procedure room, prior to the procedure, and immediately following the procedure for each laser procedure. These differences will be compared before and after the procedure, as well as between the intervention and control visits.

#### Secondary Outcomes

Average self-reported VR experience (NRS 0 [min]−10[max]) for the degree of realism, pleasure, and satisfaction with VR. These VR experience questions are assessed immediately following the laser procedure during the intervention visit.

Nurse-reported utility (binary [yes or no] questions of whether the nurse found VR-PAT to be helpful and easy to use during the procedure) is assessed immediately following the laser procedure at the intervention visit.

### Adverse Events and Oversight

Participants playing the VR-PAT game will be asked to answer the question, “Did the game make you feel not well? If yes, please explain.” Study coordinators will carefully review the collected data for any adverse events reported during the study intervention and will immediately report this information to the project manager. The principal investigators will determine how serious the event was, whether it was related, and if it was unexpected. All serious adverse events will be reported immediately to the sponsor and the IRB. During recruitment, participants will be informed of which simulator sickness symptoms to be aware of and advised that if they occur, the laser procedure can be paused to remove the headset and use the standard eye protection available. All participants will also receive a direct phone number for the research principal investigator to report adverse events.

This trial will be overseen and coordinated by the co–principal investigators and a project manager. We will have at least 1 researcher to assist with screening, recruitment, consent, and data collection. The research principal investigator and project manager meet weekly to discuss the project, including recruitment updates, challenges, and any reported adverse events. There is no specific steering committee for this study.

### Sample Size

Since this is a pilot study, the focus of the statistical analysis will be on estimating the effect sizes of VR-PAT and their 95% CI, as well as the variances of the outcome measures. These findings will facilitate sample size calculations and study power estimates for future larger-scale randomized trials. We decided on a sample of 40 participants and 2 research visits based on the grant budget and the patient volume for the 1 dermatologist collaborating on this study.

Across acute pain studies, clinically meaningful improvements are generally larger than a 1-point change on the NRS 0‐10, with systematic review evidence suggesting minimum clinically important differences of approximately 13% to 36% reduction, with a median of 23% (IQR 18%-36%), corresponding to roughly 1.4‐2.3 points on the NRS [38,39].

### Data Analysis

To achieve aim 1, composite scores will be calculated for each laser procedure, encompassing the pain score, pain medication use during the procedure, and whether rescue medication was used. The rationale for choosing the composite score as the outcome is that prior researchers have shown a significantly high positive relationship between pain medication use and self-reported pain scores [40]. From a clinical perspective, pain scores alone are influenced by the patients’ actual opioid use and may not fully reflect the treatment benefit of VR [41]. The composite score approach, therefore, provides a single, patient-centered outcome that represents overall analgesic benefit, aligning with the goal of achieving adequate pain control while minimizing opioid exposure. In our analysis, we will apply the rank-based approach by Dai et al [40] to assign weights to pain and medication use, reflecting their complementary clinical importance as measures of pain reduction [41]. In the primary analysis, the composite scores from the laser procedure with VR will be compared to those from the procedure with standard care (ie, control) using paired 1-tailed *t* tests or the Wilcoxon signed rank tests (depending on the distribution of the composite scores) due to the cross-over design. The clinical effect size will be the mean difference (if paired *t* tests are used) or median difference (if Wilcoxon signed rank tests are used) in the composite score between VR and control procedures. The SD of the difference will also be calculated.

For aim 2, the mYPAS score had 6 repeated measurements over time (T0, T1, and T2 during each of the 2 laser procedures). At the first procedure, we will compare the change in mYPAS scores from T0 to T1 and T0 to T2 between the control and VR groups using either 2-sample 1-tailed *t* tests or the Wilcoxon rank sum tests (depending on the distribution of the change in mYPAS scores). The clinical effect size will be the difference in medians of the change in mYPAS, with the confidence limits calculated by bootstrapping with replacement using 1000 repetitions. Bootstrapping has been found to provide better confidence limits with asymmetric data, such as the mYPAS score in our study, and is the recommended method. The same statistical analysis approach has been successfully used by other researchers, who reported that VR effectively reduced preoperative anxiety among 71 children aged 5 to 12 years scheduled for elective surgery [42]. At the second procedure, we will compare the mYPAS scores at T0 and T1 between the control and VR groups using the Wilcoxon rank sum tests since mYPAS scores are shown to be asymmetric [42]. The clinical effect size will be the difference in medians of mYPAS. Confidence limits will be calculated using bootstrapping again.

Linear mixed-effects models with participant-specific random intercepts will be fit on the composite scores to evaluate any modification of the effects of VR on pain by sex, age, and anxiety, with adjustment for baseline covariates and potential carryover effects of VR from the first to the second procedure. The following covariates will be included in the regression models: study group (VR vs control), period (second vs first procedure), study group by period interaction (test for carryover effect), age, age by study group interaction, sex, sex by study group interaction, procedural anxiety, procedural anxiety by study group interaction, race, ethnicity, State-Trait Anxiety Inventory for Children, 6-item State-Trait Anxiety Inventory, and average mYPAS scores between T0 and T1. We will include each of the 3 interaction terms in a separate regression model. Due to the limited sample size, testing the interaction may not be feasible. The study will then run the main linear models without interaction terms. The results will provide us with preliminary data to estimate the future study sample size and power. In case a significant carryover effect is detected, we will use only data from the first procedure to evaluate the VR effect using 2-sample *t* test or Wilcoxon rank sum tests (depending on the distribution of the composite scores).

All statistical analyses will follow the intention-to-treat principle, wherein participants will be analyzed according to their assigned study groups, regardless of compliance status or group switching during the study.

### Ethical Considerations

The NCH IRB approved this study (STUDY00002880) initially on October 17, 2022, and protocol version 6 was approved on April 1, 2025. Important protocol modifications have been communicated to study personnel by the project manager and will continue to be communicated. Written, informed consent to participate was obtained from one parent or legal guardian of all participants younger than 18 years, and from the participant if they are aged 18 years or older. Additionally, written assent was obtained from participants aged 9 years and older, as per the NCH IRB policy for written assent. This trial has been registered on ClinicalTrials.gov (NCT05645224), where detailed information about the study protocol, inclusion and exclusion criteria, interventions, outcomes, and ethical approval can be found. The trial registration process was completed before the enrollment of the first participant.

## Results

**Figure 1.**
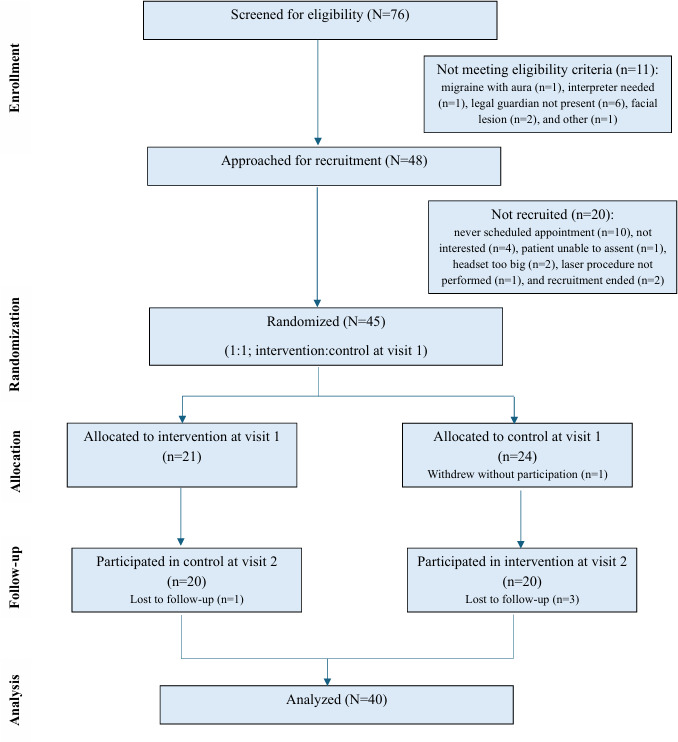
Flow diagram for a crossover, randomized clinical trial investigating the use of virtual reality during dermatologic laser procedures.

This study was funded by an intramural grant from the Clinical and Translational Science Institute at NCH (grant: IFPAWRI012023) in January 2023. Enrollment started in January 2023, and data collection was completed in November 2025. The flow of participants can be seen in Figure 1. A total of 44 patients were recruited and completed the first visit, with 40 participants completing both visits. The sample was balanced, with 40 patients using the VR intervention and 40 patients participating in the control group. Reasons for not completing both visits included discontinuing laser treatment (n=3) and a lack of interest in playing the VR game (n=1). Among those who completed both visits, the age range of the sample was 6 to 21 years at recruitment, and 55% (n=22) were female. Data analysis is expected to be finished in June 2026, with a paper to follow. Future results will be disseminated through publications in peer-reviewed journals and abstracts submitted to conferences in relevant disciplines.

## Discussion

Our VR-PAT protocols have been successfully implemented in our prior and ongoing studies, and we believe this protocol will be successful in a new setting. Prior VR research has been shown to significantly reduce self-reported pain [21-23], anxiety [10,24], and time spent thinking about pain [10,23] during painful pediatric procedures. Similar findings are anticipated in this outpatient laser study. The authors also anticipate very few reports of simulator sickness, as this study uses the same VR-PAT that has been previously used during pediatric burn dressing changes [29,31], in the emergency department [43], and during pin-pulling procedures [43]. We expect this intervention to be feasible to implement, with nurses reporting a high degree of helpfulness and ease of use, similar to our previous work [29].

The strength of this study is that laser procedures are unique clinically, in that the pain stimulus is the same for each procedure. This allows the use of a crossover study design and within-participant comparisons. Our prior VR research has been conducted during burn dressing changes, which are subject to healing over time, making each dressing change a different pain experience. Another strength of this study is providing pain and anxiety distraction during an outpatient procedure, which has historically been difficult to achieve. To maintain laser safety, shiny surfaces must be limited to avoid a reflection of the laser beam, and ocular safety requires glasses that filter the light waves and can alter the colors that patients can see. Since our VR headset passed laser safety testing, it can be a valuable tool for this patient population. Finally, we have found that the proportion of participants recruited is high among those we have approached and retention has been high among those patients who returned to the clinic for their second laser visit. Part of the retention success can be attributed to the study’s buy-in from the clinic nurses. They have helped to keep excitement high among participants by talking to them about the VR game during the laser procedures, and they have even developed an anonymized scoreboard for participants to keep track of their scores. The nurses also keep the VR headset charged and clean to ensure the study implementation runs smoothly.

This study is subject to some known limitations. First, all the participants come from the same hospital, and all the laser procedures are performed by the same physician, which limits our ability to generalize the findings. Second, since complete blinding was not possible due to the nature of the intervention, there is a chance of expectation bias among participants and clinicians. The research team attempted to manage participants’ expectations at recruitment and prior to each visit by explaining the research nature of the VR. A choice was also made not to measure participants’ expectations for the VR because we felt that this would prompt a reflection on their expectations and peak excitement. The third limitation is in evaluating the possible sample size. There has been more loss to follow-up than anticipated due to patients not following up clinically. This limits the ability to retain them in this research study. The proportion of eligible participants who were recruited is also lower than expected. This is largely related to many screened patients not ultimately scheduling a laser procedure. This will be a consideration for future studies in determining the number of physicians and/or sites needed to complete a powered study.

The future research direction, following the successful completion of this study, depends on the results but could include a fully powered efficacy study and, eventually, a multisite implementation study. In designing future implementation and dissemination work, sustainability will be a priority. This will include evaluating insurance reimbursement to cover the cost of owning and using this VR. We would follow in the footsteps of companies like AppliedVR, which was the first VR to obtain US Food and Drug Administration–authorization and established a unique Healthcare Common Procedure Coding System Level II code [44].

In conclusion, patients needing outpatient laser therapy have unique needs for pain and anxiety management, particularly in the context of ocular protection from laser damage. VR is a promising digital technology that can improve the experience of patients requiring dermatologic laser therapy. We anticipate that findings from this innovative randomized clinical trial will provide early evidence on the efficacy of VR-PAT in reducing self-reported pain and anxiety during outpatient laser procedures.

## Supplementary material

10.2196/87207 Checklist 1 SPIRIT checklist.

## Abbreviations

CONSORT: Consolidated Standards of Reporting Trials
IRB: institutional review board
mYPAS: Modified Yale Preoperative Anxiety Scale
NCH: Nationwide Children’s Hospital
NRS: Numeric Rating Scale
PDL: pulsed dye laser
RCT: randomized clinical trial
REDCap: Research Electronic Data Capture
SPIRIT: Standard Protocol Items: Recommendations for Interventional Trials
VR: virtual reality
VR-PAT: virtual reality pain alleviation therapeutics
